# Ecoepidemiology of Chagas Disease in a Biological Corridor in Southeastern Mexico: A Promising Approach to Understand the Risk of Chagas Disease

**DOI:** 10.1155/2024/4775361

**Published:** 2024-03-08

**Authors:** Ingrid Yazmin Cruz-Alegría, Nancy Gabriela Santos-Hernández, Christian Ruiz-Castillejos, Juan Felipe Ruan-Soto, Adriana Moreno-Rodríguez, Any Laura Flores-Villegas, Javier Gutiérrez-Jiménez, Luis Arturo Hernández-Mijangos, Eduardo Estanislao Espinoza-Medinilla, Dolores Guadalupe Vidal-López, José Antonio De Fuentes-Vicente

**Affiliations:** ^1^Instituto de Ciencias Biológicas, Universidad de Ciencias y Artes de Chiapas, Tuxtla Gutiérrez, Chiapas, Mexico; ^2^Facultad de Ciencias Químicas, Universidad Autónoma Benito Juárez de Oaxaca, Oaxaca, Mexico; ^3^Facultad de Medicina, Universidad Nacional Autónoma de México, Mexico City, Mexico

## Abstract

Ecoepidemiology is an emerging field that attempts to explain how biotic, environmental, and even social factors influence the dynamics of infectious diseases. Particularly in vector-borne diseases, the study under this approach offers us an overview of the pathogens, vectors, and hosts that coexist in a given region and their ecological determinants. As a result of this, risk predictions can be established in a changing environment and how it may impact human populations. This paper is aimed at evaluating some ecoepidemiological characteristics of Chagas disease in a natural reserve in southeastern Mexico that borders human settlements. We carry out a cross-sectional study in 2022 where we search insects manually and with light traps. We set traps for small mammals and bats and conducted interviews with the inhabitants living around the study site. We identified the presence of *Triatoma dimidiata* and *T. huehuetenanguensis* species with a percentage of TcI *T. cruzi* infection of 68.4% (95% CI: 66.9-69.9). Temperature and humidity were not determining factors for the probability of insect capture. Of the 108 wild mammals (Chiroptera, Rodentia, and Didelphimorphia), none was infected with *T. cruzi*. Knowledge about Chagas disease in nearby inhabitants is poor, and some characteristics were found on the periphery of dwellings that could offer a refuge for insect vectors. With this information, surveillance strategies can be generated in the study area that reduce the risk of transmission of *T. cruzi* parasite to humans, and it is expected to motivate the use of this field in future research.

## 1. Introduction

Vector-borne diseases (VBDs) represent a major threat to public health worldwide, and their emergence in new regions has put endemic and nonendemic countries on the alert [1]. An increase in the incidences of some VBDs has been registered in recent years, and forecasts of the future are not encouraging (e.g., [2]). According to the official estimates, they currently cause 700 thousand deaths per year and cause large economic losses due to work incapacity and treatment costs [3].

The typical vectors of VBDs are blood-sucking insects such as mosquitoes, ticks, fleas, and kissing bugs [4]. Insect vectors become infectious upon ingestion of the pathogen during a blood meal from an infected host. Once inside, the pathogen reproduces and/or multiplies and is then transmitted to another host such as humans to continue the cycle. An understanding of vector distribution and risk factors is important in determining the dynamics of VBDs. However, we often think that these two elements are the only ones associated with the occurrence of the disease, leaving aside the fact that the health-disease process also has social, cultural, political, economic, and environmental dimensions [5]. In order to have a thorough approach towards the control of diseases with higher occurrence and generate strategies of greater impact, the discipline of ecoepidemiology arises [6]. In particular, the ecoepidemiology of VBDs attempts to explain how biological and social factors, along with environmental alteration, influence the dynamics and potential risk of parasite transmission to human populations.

Under this premise, in this study, we evaluate some ecoepidemiological characteristics of Chagas disease in a biological corridor of southeastern Mexico, in which population growth has been evident in recent decades on the periphery. Chagas disease is one of the most important VBDs in the world due to the mortality and morbidity rate and is caused by *Trypanosoma cruzi*, a flagellated parasite naturally transmitted by the feces of kissing bugs belonging to the subfamily Triatominae (for a review, see [7]). It is well known that the loss and fragmentation of natural triatomine habitat facilitated the transmission of *T. cruzi* to humans thousands of years ago [8]. When the first civilizations settled and started activities such as cattle raising and agriculture, the resulting deforestation caused an invasion of these insects into human dwellings and the domestic cycle of infection began [9]. Nowadays, the different human activities associated with deforestation cause the propagation of the vectors to new areas (e.g., [10]), and therefore, the conservation of ecosystems is vital in order to stop this [11].

In an effort to provide information to help establish entomological surveillance strategies in the study area and to promote the comprehensive study of Chagas disease, we analyzed the triatomine species present in the area and *T. cruzi* infections, as well as infection in wild mammals, parasite genotype, awareness of Chagas disease in neighboring inhabitants, and risk factors associated with dwellings.

## 2. Materials and Methods

### 2.1. Study Area and Degradation in the Last Years

**Figure 1:**
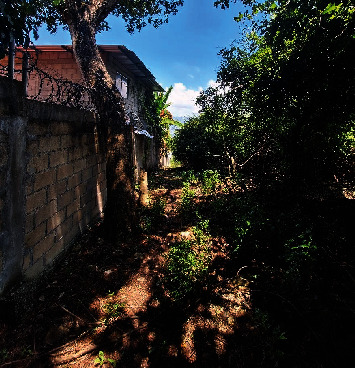
Human dwelling adjacent to the vegetation of the Cerro Mactumatza-Meseta de Copoya biological corridor.

**Figure 2:**
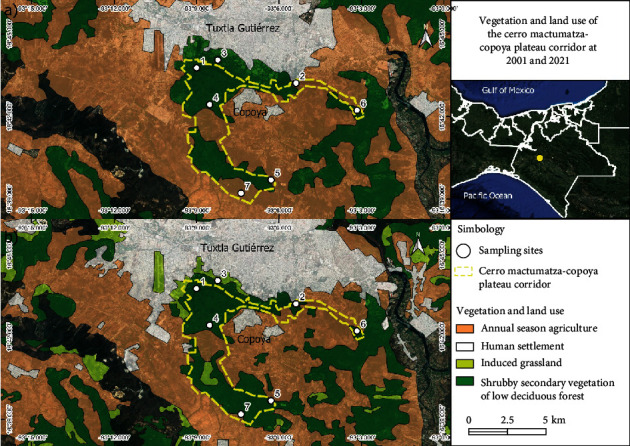
Location of the Mactumatza-Cerro de Copoya biological corridor and comparison from 2001 to 2021. The calculation of the vegetation cover area indicates a 2% growth in induced grassland. Note the growth of human settlement around the corridor.

The Cerro Mactumatza-Copoya Plateau biological corridor is located on the southern outskirts of the city of Tuxtla Gutiérrez, Chiapas, in southeastern Mexico. It is an area of 2,870 ha with an altitudinal range of 600 to 1,150 masl and a warm subhumid climate. Its vegetation is composed of medium subevergreen forest and low deciduous forest, but there are also patches of agricultural crops and pastures. It has abundant caves and springs that make it an ideal refuge for small mammals, birds, reptiles, and insects. Despite being an area subject to ecological conservation, it has not been exempt from anthropogenic alterations and contamination, since human settlements are practically adjacent to it (Figure 1). In order to know the alterations of this area, maps of 2001 and 2021 were made using vector data of land use and vegetation from Instituto Nacional de Estadística y Geografía (INEGI), obtained through the geoinformation system of Comisión Nacional para el Conocimiento y Uso de la Biodiversidad (CONABIO) (2023). The vegetation layers obtained correspond to 2001 (series II) and 2021 (series VII) at a scale of 1 : 250,000. Subsequently, the vegetation cover area was calculated within the QGis 3.22.12 software (Figure 2).

### 2.2. Triatomine Collections and Natural Infection

**Figure 3:**
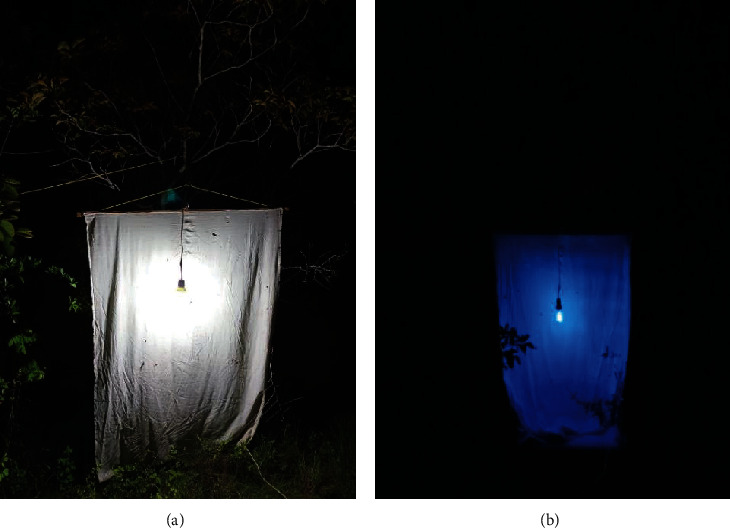
(a) White light and (b) black light traps for triatomine insect attraction.

In consultation with municipal health authorities who have been notified of possible kissing bug sightings and previous reports of infection in mammals (e.g., [12]), seven points within the biological corridor were selected for triatomine bug trapping (Figure 2). As this was an exploratory study, the points were arranged along the periphery of the corridor and near possible sighting points. During three nights per site from 6 : 00 pm to 02 : 00 h (January to May 2022), a manual search for triatomines was carried out with the help of flashlights to see between crevices, bushes, and the ground. Additionally, 20 W black and white light traps were placed on a 1.25 × 2.50 m white blanket to attract the insects (Figure 3). In total, a sampling effort of 168 hours was obtained for each capture method. The captured specimens were stored in plastic jars and transported to the laboratory for taxonomic identification. At each sampling point, data of temperature and humidity were taken with a thermohygrometer (Uni-T, UT333) as well as altitude with an e-Trex GPS (Garmin). Temperature and humidity data were recorded three times for each night: at the beginning of sampling, at the middle, and at the end of sampling. In each measurement, the device was placed 30 cm from the ground.

To detect *T. cruzi* infection, feces of the specimens were obtained by abdominal puncture and deposited on glass slides with phosphate-buffered saline (PBS) solution. The samples were observed under light microscopy at 40x (AmScope) in search of metacyclic trypomastigotes. Negative specimens were checked a second time 20 days later to rule out recent infection. Positive stool samples were then collected in tubes with 70% alcohol for DNA preservation and genotyping tests.

### 2.3. *T. cruzi* Genotyping

The ZR Fecal DNA MiniPrep Kit (Zymo Research) was used for DNA extraction from positive stool samples. For the polymerase chain reaction (PCR), three primers reported by Souto et al. [13] were used: Tc (5′CCC CCC CCC TCC CAG GCC ACA CTG 3′), TcI which amplifies a 350 base pair (bp) fragment (5′GTG TCC GCC GCC ACC ACC TTC TTC TTC CGG GCC 3′) and TcII, which amplifies a 300 bp fragment (5′CCT GCA GGC ACA CGT GTG TGTG 3′).

Cuitsi strain (TcI) and Y strain (TcII) amplifying at 350 and 300 bp, respectively, were used as positive controls. Amplification reactions were performed using a final volume of 25 *μ*l, containing 13 *μ*l of Taq Green Master Mix, 10 *μ*l of nuclease-free water, 1 *μ*l of each primer (10 *μ*M), and 1 *μ*l of positive DNA sample. Amplification conditions were as follows: 5 minutes at 94°C followed by 30 cycles of 40 seconds at 94°C, 40 seconds at 61°C, and one minute at 72°C ending with 5 minutes at 72°C. The amplified products were visualized on 3% agarose gels using a 50 bp Invitrogen™ Ladder DNA marker.

### 2.4. Small Mammal Sampling and Natural Infection

Small wild mammals were captured at the sampling sites using 20 Sherman and Tomahawk traps. As bait, a mixture of vanilla with oatmeal and peanut butter was used. To capture bats, two mist nets (10 meters and 12 meters) were placed near water reservoirs and fruit trees with abundant vegetation. The traps were set on the same nights as insect trapping but placed at least 50 m from the insect search points. Traps for terrestrial and flying mammals were kept from dusk until 8 hours later. In this way, a small mammal capture effort of 420 trap nights was obtained. While for the capture of bats, a capture effort of 3,696 m net/night was obtained.

Captured terrestrial mammals were anesthetized with ketamine (50 mg/kg) for handling and posterior distal blood extraction through a small incision in the tail. Bats were carefully removed from mist nets and handled with bait gloves for cardiac blood collection with a U-100 1 ml insulin syringe (27 G × 13 mm). The collected blood from all mammals was preserved in BD Microtainer® tubes with K2EDTA for subsequent DNA extraction. For DNA extraction and *T. cruzi* detection, we used the same procedure for genotyping as mentioned above.

### 2.5. Knowledge and Risk Factors about Chagas Disease

An analysis of the knowledge about Chagas disease was carried out among the people living in the immediate vicinity of the sampling sites. A total of 216 people were interviewed at the seven sampling points. Men and women over 18 years of age were included, and only one interview per household was conducted. As a data collection instrument, a semistructured interview [14] was used, consisting of (a) questions to obtain data on the knowledge about Chagas disease, (b) questions to obtain data on knowledge about the vector (identification and biology), and (c) risk factors associated with vector infestation and colonization.

### 2.6. Statistical Analysis and Bioethical Considerations

In order to evaluate differences between male and female insect captures, as well as the percentage of infection between species, the chi-square test included in the R package was used. The information collected from the surveys was sorted in Epi Info™ version 7.1 and is shown as descriptive statistics. The handling of wild animals was performed in accordance with the stipulations of the General Wildlife Law. The capture of animals was approved by the Mexican Ministry of Environment and Natural Resources through collection permit SEMARNAT 07/K5-0243/08/22. No specimens were killed or removed from the sampling sites. Prior to the application of the interviews with the residents, the local authorities were informed of the study's aim. Likewise, informed consent was obtained from each participant. This research was endorsed by the Biosafety and Research with Human Subjects Subcommittee (SBISH/02/2023) of the Ethics Committee of the Institute of Biological Sciences of the University of Sciences and Arts of Chiapas.

## 3. Results

**Figure 4:**
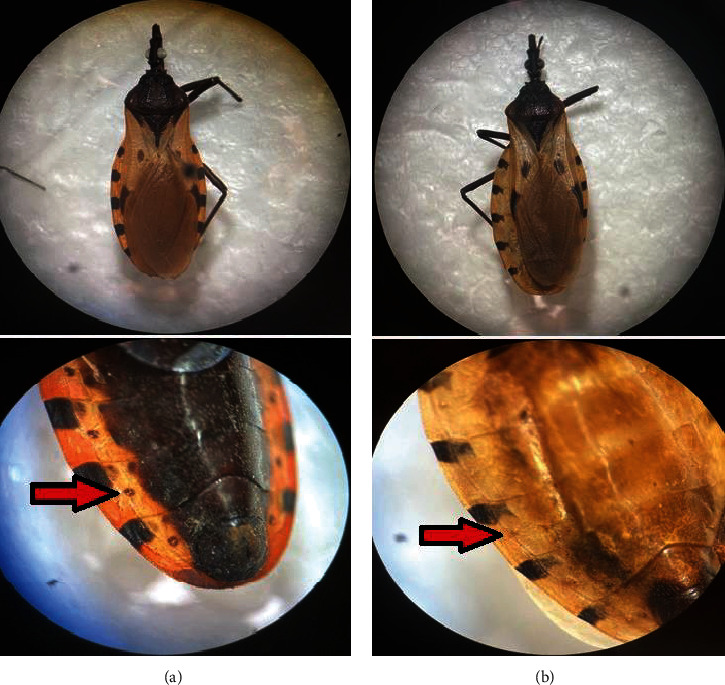
Triatomine species found in the biological corridor Cerro Mactumatza-Meseta de Copoya. (a) *T. dimidiata* and (b) *T. huehuetenanguensis*. Note the differences in the connexall spiracles in ventral view (below), where in *T. huehuetenanguensis*, they are not very pronounced and are concolorous with the rest of the integument unlike *T. dimidiata* [15].

A total of 38 specimens of adult stage triatomines were collected throughout the sampling sites. Although captures were made at all sites, sites 1 and 4 concentrated 53% of the specimens. These sites are those closest to human settlements, and significant differences were found with respect to the other sampling points (*X*^2^ = 4.25; *p* = 0.037). Twenty-three of these were identified as *T. huehuetenanguensis* [15] and 15 as *Triatoma dimidiata* [16] (Figure 4). Interestingly, for *T. dimidiata*, there was a higher capture of female specimens (73.2%; *X*^2^ = 4.4; *p* = 0.023), while in *T. huehuetenanguensis*, males were the dominant sex (78.2%; *X*^2^ = 3.5; *p* = 0.012). The average temperature (To) of the collection sites was 24.55 ± 1.30 (SD), the altitude (Alt) was 776.69 m ± 98.26 (SD), and the average humidity (Ho) was 69.62% ± 11.29 (SD). Pearson's correlation analysis indicated that there is no relationship between environmental variables and the probability of capture individuals of both species (*R*^2^: To = 0.286, Alt = 0.016, and Ho = 0.192).

The total *T. cruzi* infection prevalence was 68.4% (26/38), but *T. huehuetenanguensis* had a significantly higher infection rate (78.2%) than *T. dimidiata* (53.3%) (*X*^2^ = 0.017; *p* = 0.024) (Table 1). No significant differences were found between the sex of the specimen and the occurrence of infection (*X*^2^ = 1.78; *p* > 0.645) or the capture site (*X*^2^ = 12.41; *p* > 0.245). From all 26 of the *T. cruzi*-positive specimens, genetic analysis with the miniexon gene showed the amplification of 350 bp products, revealing the circulation of the TcI lineage only.

Additionally, 108 wild mammals were captured, belonging to three different orders: Chiroptera, Rodentia, and Didelphimorphia. Most of the captured specimens corresponded to Chiroptera (100/108) and to a lesser extent Rodentia (6/108) and Didelphimorphia (2/108) (Table 2). Bat captures were made at all sampling points, without significant differences between points (*X*^2^ = 1.49; *p* > 0.6835). However, there were only captures of terrestrial mammals in sites 1 and 3 (5 and 3 specimens, respectively). Although blood samples were collected from all the species captured, none of them tested positive for *T. cruzi*.

From the interview data, it was observed that only 8.8% of the respondents said they were familiar with Chagas disease and only half of them knew how it is transmitted. Other results of the interviews are shown in Table 3, where 47.2% know about the insect vector, but only 20.4% of them consider it dangerous.

Although most of the surrounding houses are made of materials that do not favor the infestation of vector insects (mainly concrete), some characteristics such as the presence of animals inside the dwellings, the lack of window screens, the poor fumigation of the houses, and the presence of materials in the peridomicile (Table 4) may offer a latent risk for the infestation of triatomine insects.

## 4. Discussion

A little more than 110 years after its discovery, our best weapon to combat Chagas disease continues to be prevention focused on the insect vector. Although health authorities in endemic countries carry out constant vector control campaigns in areas of insect distribution, the fact is that many areas remain unexplored. The ecoepidemiological approach in potential areas of Chagas disease can help us to have a more complete view of the situation and to intervene in a timely manner to prevent transmission to humans.

This paper represents the first report of triatomine insects in the study area, but wild circulation of *T. cruzi* had already been reported in small mammals since the 1990s' [17] and was recently updated by another study that found a prevalence of 28.57 in small mammals and 16.36% in bats [12]. The largest number of mammalian specimens captured in this study was bats, which are abundant in this area due to the caves that offer a perching site. Although some triatomines can inhabit caves (e.g., [18]), little has been studied about the dynamics of *T. cruzi* in these ecotopes. On the other hand, the remnants of low deciduous forest in this reserve provide different refuges for small mammals such as cracks in rocks and trees, foliage, or fallen trunks, which can also be exploited by triatomine insects. However, we did not find any infected mammals despite making the captures in the same period of the year as the previous work, as it is suggested that the distribution of *T. cruzi* can become grouped in space and time [19]. This has also been observed in insect vectors, where the abundance and frequency of infection varies according to the season of the year [20].

Here, we show records of *T. huehuetenanguensis* and *T. dimidiata* infected with *T. cruzi*. Environmental variables measured in the study area suggest favorable conditions for several species of triatomines, including *T. dimidiata* (e.g., [21]). In addition, high thermal tolerance capacity has been evidenced in members of this group [22]. Both species were found to be infected by TcI, the predominant genotype in Central and North America and which is related to the domestic and wild cycle of Chagas disease [23], suggesting a great plasticity to infect different triatomine species. The species *T. huehuetenanguensis* was recently described in Guatemala [15], and its epidemiological relevance in the transmission of *T. cruzi* to humans is largely unknown, although it could represent an important vector in the study area given the high percentage of infection found. However, it is not yet known which natural refuges this species is capable of exploiting. Some biological parameters measured in the laboratory seem to indicate a high vector potential of *T. huehuetenanguensis* [24]. Furthermore, some individuals have been collected inside dwellings, but none infected with *T. cruzi* [25]. In this sense, it is necessary to continue investigating their role in the epidemiology of Chagas disease.

On the contrary, *T. dimidiata* is widely distributed and is considered the main vector in southeastern Mexico, Central America, and northern South America [26]. It has a diverse ecological niche where it exploits different natural refuges such as caves, rocks, tree holes, and vertebrate nests [27], which are found in our study site. This capacity is closely linked to the extensive genetic variation shown [27]. Furthermore, in wild environments, it feeds on a wide variety of animals, including synanthropic ones that may act as links to the domestic cycle [28]. Although *T. dimidiata* was the least abundant species in this study, its ability to invade and colonize dwellings is well known [28], representing a latent risk to surrounding dwellings. Deforestation and change of land use in this biological corridor may lead to the displacement of wild individuals towards dwellings in search of food sources or simply be attracted by artificial light as has been observed in other studies [29, 30]. The interviews conducted evidenced that the inhabitants store materials in the peridomicile that could provide shelter for insects, such as wood or block shelter and the presence of some barnyard animals. Although we do not know the mechanisms underlying the invasion and colonization of dwellings, the invasion of the peridomicile could be the origin of this process. Fortunately, the interior of the interviewed dwellings are made of materials that would make the establishment of domestic populations difficult, such as concrete floors, walls, and roofs (see [31]).

On the other hand, community participation can contribute to governmental vector control efforts and prevent the invasion of human dwellings. These participations in some cases have yielded positive results (e.g., [32]) but in other cases have been insufficient [33]. Analysis of knowledge and perceptions prior to strategy design is critical to the success of a health intervention (e.g., [34]). Our study found that most participants have poor knowledge about Chagas disease or have not heard of it. A previous study in rural areas in the same region of Mexico also found low levels of knowledge about Chagas disease [35]. This highlights the importance of continuing education campaigns on the disease, especially in highly endemic areas such as southeastern Mexico. Likewise, it was found that half of them know the insect vector and very few consider it dangerous. It is important to mention that the similarity of triatomines with other nonharmful reduvids can lead to confusion and bias information (See [36]).

Finally, only a third of the homes surveyed carry out periodic fumigation to eliminate harmful insects. However, some studies have shown that, at least in *T. dimidiata*, intradomiciliary populations recover significantly after house fumigation campaigns (e.g., [33, 37]). In this sense, systematic house fumigation appears to be insufficient to eradicate domiciliary insect populations or prevent infestation. Side-by-side alternatives such as home improvement and the use of window screens may be a feasible option to avoid the above [32], but the cost of these improvements may be an issue in extremely poor areas, and other options should be considered.

## 5. Conclusions

A successful control strategy for Chagas disease requires first a complete overview of the problem in a specific region. Our study reveals the presence of insect vectors infected with TcI in the study area and some factors that may endanger the public health of nearby inhabitants. However, we were unable to analyze the blood-feeding source of the captured insects, which would give us an idea of the mammals involved in the *T. cruzi* transmission cycle in this reserve. In general, this study highlights the importance of having an entomological surveillance program in which the community takes part, especially when one of the vectors found is among the most important in the epidemiology of Chagas disease. The analysis of the ecoepidemiological components in future studies could provide us with information that would allow for a thorough intervention and decision-making focused on the problem. For example, combining vector surveillance and health education could be a successful strategy to prevent the spread of wild insects to urban areas sites such as ours. As we know, Chagas disease has been gaining ground in urban areas for different reasons, and it is everyone's task to prevent this from continuing. Although it may be difficult to encourage the continuous participation of a community in health campaigns, we must look for ways to promote self-care in community health.

## Figures and Tables

**Table 1:** Specimen triatomines captured in the biological corridor Cerro Mactumatza-Meseta de Copoya.

Specie	Sex	Captured specimens	Stage	Specimens infected with *T. cruzi*
*T. huehuetenanguensis*	Female	5	Adults	3
	Male	18	Adults	15

*T. dimidiata*	Female	11	Adults	6
	Male	4	Adults	2

Total		38		26

**Table 2:** Mammals captured in the biological corridor Cerro Mactumatza-Meseta de Copoya. No individual tested positive for *T. cruzi.*

Order	Family	Species	Number of specimens captured
Chiroptera	Phyllostomidae	*Artibeus jamaicensis*	40
		*Artibeus lituratus*	31
		*Glossophaga mutica*	14
		*Desmodus rotundus*	7
		*Choeroniscus godmani*	1
	Mormoopidae	*Pteronotus mesoamericanus*	6
	Emballonuridae	*Balantiopteryx plicata*	1
		Total	100

Rodentia	Cricetidae	*Peromyscus mexicanus*	4
	Heteromyidae	*Liomys pictus*	2
		Total	6

Didelphimorphia	Didelphidae	*Didelphis virginiana*	1
		*Didelphis marsupialis*	1
		Total	2

		Total	108

**Table 3:** Knowledge about Chagas disease in inhabitants living near the biological corridor Cerro Mactumatza-Cerro de Copoya (*N* = 216).

Survey questions	%
About Chagas disease	
Knows what Chagas disease is	8.8
Knows how it is transmitted	4.2
Knows its effects	3.2
Knows about its treatment	1.4
Associates the disease with some symptoms	6.9

About the insect vector	
Knows the insect	47.2
Knows the importance of the insect	5.1
Considers the insect dangerous	20.4
Recognize it by some characteristic	31.5
Recognize it by some name	31.9
Associates the presence of the vector with animals	2.8
Associates the presence of the vector with a particular season	37.1

**Table 4:** Characteristics of dwellings that have been associated with risk of infestation by kissing bugs.

Risk factor	%
Homes with tile roof	0.9
Homes with adobe walls	2.3
Dwellings with dirty floors	1.9
Presence of animals in the dwelling	74.1
Presence of materials in the peridomicile	63.2
No frequent cleaning of the peridomicile	2.31
No mosquito netting screens on the windows	50.5
Lack of fumigation of the dwelling	38.4
Firewood transported to the house	16.67

## Data Availability

The data used to support the findings of this study are included within the article. If anyone is interested in the raw data (Excel), it is available from the corresponding authors upon request.
